# Validation of Peak Growth Hormone Levels After Clonidine and Glucagon Stimulation Tests in Children With Severe Short Stature

**DOI:** 10.7759/cureus.100084

**Published:** 2025-12-25

**Authors:** Sridhar Subbiah, Rameez Raja, Dhivya Shanmugam, Sreenivasan Palaniappan, Sreekumar Shanmugam, Muthu Aravind Kumar

**Affiliations:** 1 Endocrinology, Madurai Medical College, Madurai, IND; 2 Endocrinology, Diabetes and Metabolism, Kauvery Hospital, Chennai, IND

**Keywords:** clonidine stimulation test, glucagon stimulation test, growth hormone deficiency, growth hormone provocation test, short stature

## Abstract

Introduction

This study evaluated the concordance and temporal dynamics of growth hormone (GH) responses during the clonidine stimulation test (CST) and glucagon stimulation test (GST) in children being evaluated for growth hormone deficiency (GHD), and identified optimal sampling time points that may allow protocol simplification without compromising diagnostic agreement.

Methods

This prospective study included 55 children who were subjected to CST and GST at a one-week interval. Blood samples for GH were taken at different time intervals following each provocative test per standard protocols. GH levels were estimated by the electrochemiluminescence immunoassay (ECLIA) method. A peak GH level of less than 8 ng/mL was considered diagnostic of GHD.

Results

Among 55 children, 41 were diagnosed with GHD and 14 with idiopathic short stature (ISS). Overall, peak GH levels were noted at 0, 60, and 90 minutes after CST and 0, 120, and 180 minutes after GST in more than 98% of the study cohort. The sensitivity of CST and GST in diagnosing GHD was 87.8% and 90.2%, and the specificity was 85.5% and 100%, respectively. The combined provocative tests (CST and GST) had an area under the curve (AUC) of 1.000, outperforming the individual tests, which had an AUC of 0.914 for CST and 0.987 for GST, to diagnose GHD and ISS.

Conclusions

CST and GST show high concordance in the evaluation of GHD using modern ECLIA-based assays, with GST demonstrating relatively higher specificity. Optimization of sampling time points permits protocol simplification without compromising diagnostic agreement, improving clinical utility.

## Introduction

The diagnosis of growth hormone deficiency (GHD) in children and adolescents requires a detailed clinical and biochemical assessment, followed by radiological confirmation [1]. Growth hormone (GH) provocative tests play an essential role in the diagnosis of GHD, and at least two provocative tests are required to confirm short stature due to GHD as per consensus guidelines [1,2]. The insulin tolerance test (ITT), although considered the gold standard, is infrequently used nowadays due to its several risks [3]. Other GH stimulants, such as clonidine, glucagon, arginine, levodopa, growth hormone-releasing hormone (GHRH), and recently macimorelin, are used in various clinical settings, depending on their availability in various regions [4,5]. The clonidine stimulation test (CST) is the most commonly performed GH provocative test nowadays. The glucagon stimulation test (GST), although it offers several advantages over other tests, has been poorly studied in children to date. The objectives of this study were to assess the agreement and timing of peak GH responses between CST and GST in children undergoing evaluation for GHD, and to determine whether selected sampling time points could be safely omitted, thereby optimizing test protocols and improving clinical feasibility while maintaining diagnostic concordance.

## Materials and methods

This prospective study was conducted in children with severe short stature and suspected GHD. The study was performed at a multispecialty tertiary care referral government hospital in southern India between September 2022 and March 2025, following Institutional Ethical Committee (IEC) approval. Severe short stature was defined as height less than three SDs below the mean according to the WHO 2006 and IAP (Indian Academy of Pediatrics) 2015 growth charts [2,6]. All syndromic short stature cases, including Turner syndrome, Noonan syndrome, and Prader-Willi syndrome, were excluded from the analysis. Short stature due to pituitary neoplasm, chronic systemic illness, and skeletal dysplasia was also excluded from the study. Sample size was calculated using sensitivity, specificity, positive predictive value (PPV), and negative predictive value (NPV) as diagnostic accuracy parameters, based on values reported in previous literature (expected sensitivity of 80%, precision ±10%, and 95% CI), using Epi Info StatCalc (version 7.2) (Centers for Disease Control and Prevention, Atlanta, GA).

A detailed history, including birth history, family history, and degree of consanguinity, was elicited. The height, height standard deviation score (SDS), weight, weight SDS, body mass index (BMI), BMI SDS, target height, and target height SDS were calculated. Clinical features such as frontal bossing, midfacial hypoplasia, cleft lip, cleft palate, single central incisor, congenital squint, and nystagmus were examined. Pubertal status was assessed using gender-specific Tanner staging. Bone age was assessed using the Greulich-Pyle chart. The bone age to chronological age ratio was calculated, and a ratio of less than 0.8 was considered a delayed bone age [7]. All hormonal investigations, including GH, insulin-like growth factor 1 (IGF-1), thyroid-stimulating hormone (TSH), free thyroxine (fT4), cortisol, prolactin, luteinizing hormone (LH), follicle-stimulating hormone (FSH), testosterone, and estradiol, were measured using a Roche Cobas e411 electrochemiluminescence immunoassay (ECLIA) analyzer. The functional and analytical sensitivities of the GH assay were 0.05 ng/mL and 0.03 ng/mL, respectively. The intra-assay coefficient of variation (CV) was <4.5%, and the inter-assay CV was <6.5%.

Associated pituitary hormone deficiencies were corrected before GH provocative tests. Sex-steroid priming was performed only in prepubertal children (Tanner stage I) to reduce false-positive GHD diagnoses. Priming was administered to boys aged ≥11 years and girls aged ≥10 who were Tanner stage I. Children who had already entered puberty (Tanner stage ≥2) did not undergo priming [8]. Boys received intramuscular testosterone enanthate 50 mg one week prior, and girls received oral estradiol valerate 2 mg once daily for three days prior to GH stimulation testing. All participants underwent the CST first, followed by the GST after a washout period of at least one week. Blood samples were collected via a heparinized scalp vein set. All stimulation tests were performed after an overnight fast of at least eight hours, between 7:00 and 8:00 AM. Samples were centrifuged within one hour of collection at 3,000 rpm for 10 minutes, and serum was separated and stored at -20°C until analysis. For the CST, clonidine tablets were administered at a dose of 5 μg/kg, up to a maximum of 150 μg, and blood samples for GH were drawn at 0, 30, 60, 90, and 120 minutes [1]. For the GST, glucagon was given intramuscularly at a dose of 0.03 mg/kg up to a maximum dose of 1 mg, and blood samples were drawn at 0, 30, 60, 90, 120, 150, and 180 minutes. All blood samples were collected via a heparinized scalp vein set inserted into a forearm vein. GHD was diagnosed when a peak GH level of less than 8 μg/L was achieved after both stimulation tests [9,10]. Idiopathic short stature (ISS) was diagnosed when the peak GH level exceeded 8 μg/L during any GH provocative test, after excluding other causes of short stature. MRI of the pituitary was performed in all cases and was reported by two independent radiologists. All provocative tests were performed as inpatient procedures after obtaining informed written consent from the parents.

Statistics

All statistical data were analyzed using SPSS version 26 (SPSS Inc., Chicago, IL), and descriptive statistics were computed. Serum GH at different time intervals was presented as mean±SD, median, and interquartile range (IQR). Categorical variables, including demographic and clinical characteristics, were compared using the chi-square test or Fisher’s exact test based on expected cell counts. For all quantitative parameters, mean values were compared using paired or unpaired Student’s t-tests. The CI was set at 95%. Receiver operating characteristic (ROC) curve analysis was performed using MedCalc Statistical Software (version 16.0) (MedCalc Software Ltd., Ostend, Belgium). ROC analysis was conducted to assess the diagnostic accuracy of CST and GST peak GH values in differentiating between GHD and ISS. The area under the ROC curve (AUC) and corresponding 95% CI were computed to quantify the discriminatory power of each test. The optimal cutoff values were determined using Youden’s index (J = sensitivity + specificity - 1) to maximize diagnostic performance. Sensitivity, specificity, PPV, and NPV were calculated for each test at the optimal threshold. To compare the performance of CST and GST, a pairwise comparison of ROC curves was performed using DeLong’s test for correlated AUCs [11]. The significance level was set at p<0.05. Incorporating CST and GST peak GH values as independent variables, an ROC curve was generated for the combined model. The AUC of the combined model was compared with the AUCs of individual CST and GST to assess whether a dual-test approach improved diagnostic accuracy.

## Results

Clinical, hormonal, and radiological characteristics of study cohorts

Among 55 severely short-statured children, 29 (52.7%) were male. Forty-one were diagnosed with GHD, and 14 were diagnosed with ISS. Among the 41 children with GHD, 31 had isolated growth hormone deficiency (IGHD) and 10 had multiple pituitary hormone deficiency (MPHD). The mean chronological age at diagnosis was 11.5±3.8 years, and a history of consanguinity was present in 26 (47.3%) of the study cohort. Four (7.3%) participants had breech presentation at delivery, and all of them had GHD. Twenty subjects were primed with estradiol valerate before the GH provocative tests. MRI showed a normal pituitary, hypoplastic pituitary, pituitary stalk interruption syndrome (PSIS), partial empty sella, and septo-optic dysplasia in 15 (27.3%), 25 (45.5%), 10 (18.2%), four (7.2%), and one (1.8%) of the patients, respectively [12].

**Figure 1:**
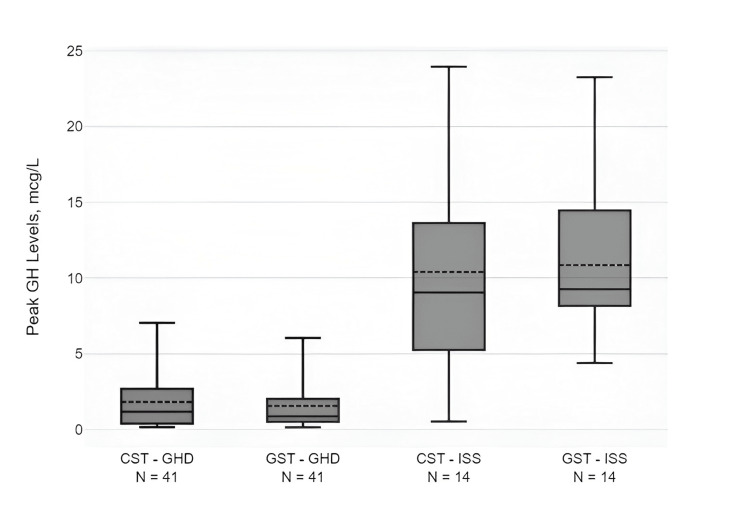
Box-and-whisker plot showing mean (dashed line within the box), median (thick line within the box), and IQR of peak GH levels (μg/L) after CST and GST in children with GHD and ISS. CST, clonidine stimulation test; GST, glucagon stimulation test; GHD, growth hormone deficiency; ISS, idiopathic short stature; GH, growth hormone; IQR, interquartile range

Clinical, hormonal, and radiological characteristics of the GHD and ISS groups are compared in Table 1. The mean peak GH level following CST and GST was 1.81±1.89 μg/L and 1.55±1.55 μg/L, respectively, in the GHD cohort. Box-and-whisker plots of the mean, median, and IQR of peak GH levels after CST and GST in children with GHD and ISS are shown in Figure 1. Table 2 compares the mean and median GH peaks at 0, 30, 60, 90, and 120 minutes following CST, as well as at 0, 30, 60, 90, 120, 150, and 180 minutes following the GST for the entire 55-subject cohort. Overall, peak GH levels were noted at 0, 60, and 90 minutes after CST and at 0, 120, and 180 minutes after GST in more than 98% of the study cohort.

**Table 1:** Clinical, hormonal, and radiological characteristics of GHD and ISS study groups. Continuous variables are presented as mean values and compared using the unpaired Student’s t-test. Categorical variables were analyzed using the chi-square test or Fisher’s exact test (when cell counts were less than five). Test statistics (t or χ²) are shown in the final column; Fisher’s exact test does not yield a test statistic and is indicated by a "-." GHD, growth hormone deficiency; ISS, idiopathic short stature; SDS, standard deviation score; BMI, body mass index; BA, bone age; CA, chronological age; IGF-1, insulin like growth factor-1; CST, clonidine stimulation test; GST, glucagon stimulation test; GH, growth hormone *Significant P-value

Characteristics		GHD	ISS	GHD vs. ISS	Test statistic (t/χ²)
		n=41	n=14	P-value	
Age in years, mean ± SD		11.7±4.0	10.8±3.2	0.403	t=0.85
						Male, n (%)		23 (56.1%)	6 (42.8%)	0.392	χ²=0.73
History of consanguinity, n (%)		19 (46.3%)	7 (50.0%)	0.812	χ²=0.056
Breech presentation, n (%)		4 (9.8%)	0	0.22	-
Birth weight (kg), mean ± SD		2.83±0.35	2.69±0.15	0.154	t=2.07
						Height SDS, mean ± SD		-4.18±1.19	-3.48±0.66	0.042^*^	t=-2.73
Weight SDS, mean ± SD		-3.37±1.17	-3.29±0.72	0.811	t=-0.30
BMI SDS, mean ± SD		-1.47±1.27	-2.05±1.37	0.154	t=1.39
Target height SDS, mean ± SD		-1.38±0.86	-1.36±0.81	0.939	t=-0.08
BA/CA ratio, mean ± SD		0.6±0.14	0.63±0.15	0.499	t=-0.66
IGF-1 SDS, mean ± SD		-2.2±0.3	-1.1±0.2	<0.001^*^	t=-15.48
CST GH peak (mcg/L), mean ± SD		1.81±1.89	10.39±6.69	<0.001^*^	t=-4.73
GST GH peak (mcg/L), mean ± SD		1.55±1.55	10.85±5.17	<0.001^*^	t=-6.63
	Normal pituitary, n (%)	9 (21.9%)	6 (42.9%)	0.129	χ²=2.30
MRI pituitary	Pituitary stalk interruption syndrome, n (%)	10 (24.4%)	0	0.041	-
	Hypoplastic pituitary, n (%)	18 (43.9%)	7 (50.0%)	0.692	χ²=0.16
	Partial empty sella, n (%)	3 (7.3%)	1 (7.1%)	0.982	χ²=0.00047
	Septo optic dysplasia, n (%)	1 (2.5%)	0	0.555	-

**Table 2:** Mean ± SD, median, and IQR of serum GH levels (μg/L) at various time intervals during CST and GST in the study cohort. GH, growth hormone; NA, not applicable; CST, clonidine stimulation test; GST, glucagon stimulation test; IQR, interquartile range

CST							
Time (minutes)	0	30	60	90	120	150	180
GH (mcg/L), mean ± SD	1.37±2.57	1.36±2.01	2.68±4.13	3.33±4.60	1.92±2.03	NA	NA
GH (mcg/L), median	0.56	0.7	0.98	1.13	0.9	NA	NA
GH (ng/mL), IQR	0.30-1.06	0.30-1.39	0.44-2.55	0.40-4.68	0.30-3.05	NA	NA
GST							
Time (minutes)	0	30	60	90	120	150	180
GH (mcg/L), mean ± SD	0.91±0.96	0.88±0.98	1.07±1.16	1.42±1.8	2.3±3.9	1.73±1.84	2.51±3.90
								GH (mcg/L), median	0.56	0.5	0.56	0.7	0.79	0.74	0.91
GH (mcg/L), IQR	0.3-1.08	0.22-1.2	0.33-1.4	0.29-1.8	0.4-2.8	0.37-2.7	0.4-2.33

ROC analysis was performed to assess the diagnostic accuracy of peak GH levels from CST and GST to correctly diagnose GHD and ISS. The AUC for the peak GH value was 0.914 (95% CI: 0.806-0.972) for CST, indicating excellent discriminatory ability. The optimal cutoff value for CST, determined using the Youden index, was ≤4.45 ng/mL (range: 1.48-6.4 ng/mL), resulting in a sensitivity of 87.8% and a specificity of 85.5%. The PPV and NPV for CST were 94.6% and 70.57%, respectively.

For GST peak GH values, the AUC was 0.987 (95% CI: 0.911-1.000), suggesting near-perfect diagnostic accuracy. The optimal cutoff value for GST was ≤3.62 ng/mL (range: 1.87-6.04 ng/mL), with a sensitivity of 90.24% and specificity of 100%. The PPV and NPV for GST were 100% and 77.7%, respectively, indicating that GST had no false positives in this cohort.

ROC analysis was performed by combining CST and GST peak GH values. The ROC analysis of the combined model yielded an AUC of 1.000, indicating perfect discrimination between GHD and ISS cases. The combined model outperformed CST (AUC=0.914) and GST alone (AUC=0.987), suggesting that using both stimulation tests together improves diagnostic accuracy up to 100%.

Comparison of CST and GST ROC curves

**Figure 2:**
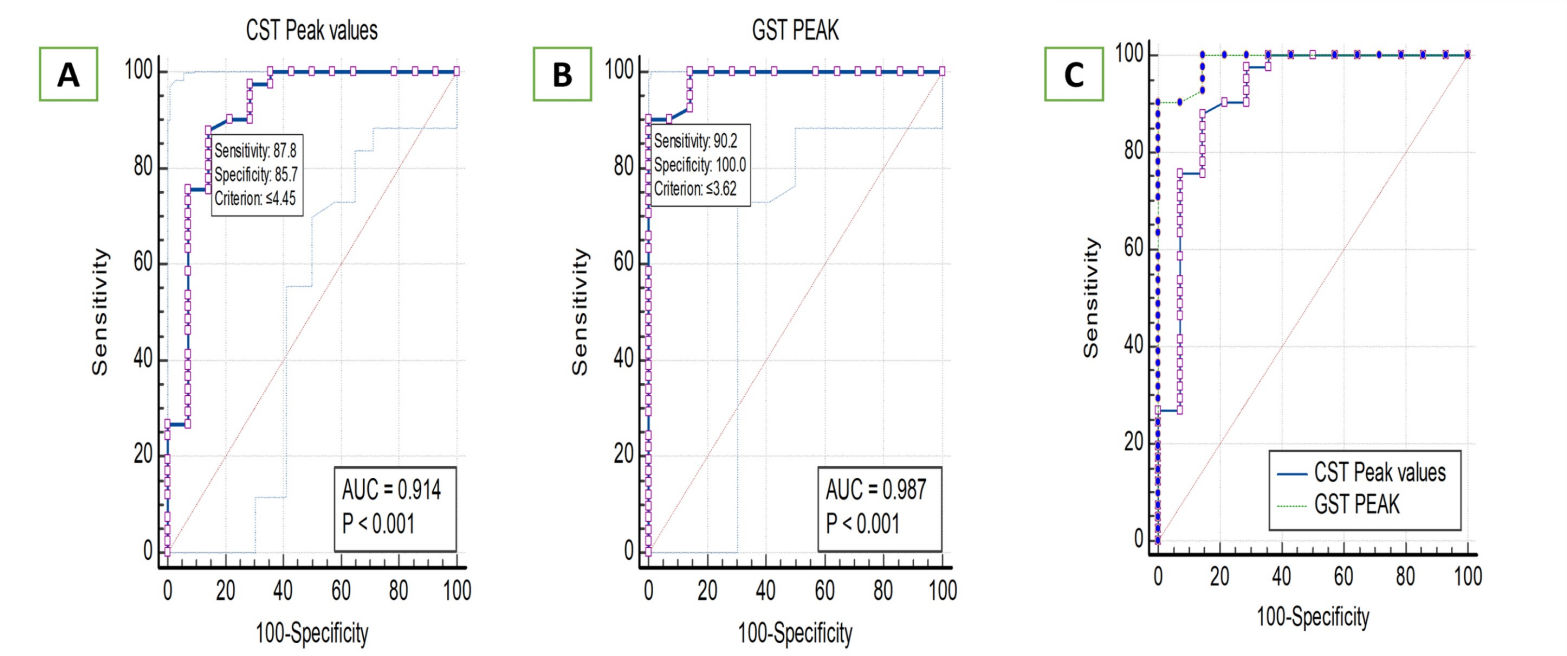
A: Diagnostic performance of CST in all children with severe short stature. B: Diagnostic performance of GST in all children with severe short stature. C: Diagnostic performance of combined CST and GST in all children with severe short stature. CST, clonidine stimulation test; GST, glucagon stimulation test

Pairwise comparison of the ROC curves for CST and GST showed a difference in AUC of 0.0732 (95% CI: -0.0384 to 0.185). DeLong’s test for statistical significance yielded a p-value of 0.1986, indicating that the diagnostic performance difference between CST and GST was not statistically significant. However, the combined model achieved superior performance compared to individual tests (Table 3), supporting the potential benefit of integrating both CST and GST in clinical evaluation. The diagnostic performance of CST, GST, and the combined model in the study cohort is depicted in Figure 2A, Figure 2B, and Figure 2C.

**Table 3:** Diagnostic performance of CST, GST, and the combined model in the study cohort. AUC, pairwise comparison: the difference in AUC between CST and GST was 0.0732 (95% CI: -0.0384 to 0.185, p=0.1986, DeLong’s test), indicating no statistically significant difference between the two tests; calculated based on the Youden index. GHD, growth hormone deficiency; CI, confidence interval; AUC, area under the curve; GH, growth hormone; CST, clonidine stimulation test; GST, glucagon stimulation test; PPV, positive predictive value; NPV, negative predictive value

Variable	AUC (95% CI)	Sensitivity (%)	Specificity (%)	Cutoff value (ng/mL)	NPV (%)	PPV (%)
CST peak GH	0.914 (0.806-0.972)	87.8	85.5	≤4.45 (1.48-6.4)	70.57	94.6
GST peak GH	0.987 (0.911-1.000)	90.24	100	≤3.62 (1.87-6.04)	77.7	100
CST+GST (combined model)	1	100	100	-	100	100

## Discussion

A random GH level does not help diagnose GHD, given its episodic and pulsatile secretion. Hence, the GH provocative test is an essential tool for diagnosing GHD in all cases of short stature, unless it is associated with other pituitary hormone deficiencies or PSIS [1]. In addition, GH provocative tests are also imperative for the treating pediatrician or endocrinologist to determine the appropriate dose and to predict the response to GH therapy. Finally, GH provocative tests are helpful to confirm the diagnosis of ISS.

Estimating GH accurately is challenging due to its heterogeneous molecular structure, complex isoforms, and diverse GH-binding proteins [13]. Different immunoassays, ranging from radioimmunoassay (RIA), immunoradiometric assay (IRMA), and enzyme-linked immunosorbent assay (ELISA) to, recently, chemiluminescence immunoassay (CLIA), have been used in clinical laboratories over different periods to estimate GH levels with various sensitivity, specificity, and CV. Recently, ECLIA, an ultrasensitive assay with a GH detection limit of 0.030 μg/L, has proven more sensitive and highly specific than older assays with lower detection (0.3-0.5 μg/L) [14,15]. The diagnostic cutoff level of GH varies depending on the immunoassay used. The cutoff for GH levels after provocative agents in RIA and ELISA is 5 μg/L, whereas in ECLIA, the cutoff is 7.77 μg/L [9]. In addition, diagnostic cutoff values for different provocative tests vary across various ethnicities and consensus guidelines (6.7 μg/L in France, 8.0 μg/L in Germany, and 10 μg/L in the United States [1,2,10]). Despite various limitations of provocative tests, including the potency of GH provocative agents, assay methodology, sex-steroid priming, coexisting MPHD, and different ethnicities, most guidelines recommend the same cutoff level for all GH provocative tests [10]. In the present study, we used a cutoff of 8 μg/L to diagnose or exclude GHD following both provocative tests [9,10]. The rationale for choosing the 8 μg/L cutoff reflects the transition from older RIAs (cutoff 10 μg/L) to modern, more specific chemiluminescent assays, which yield lower GH values for the same physiological response. This revised threshold may improve diagnostic accuracy while reducing overdiagnosis of GHD.

Various pharmacological agents currently available for provocative tests include insulin, clonidine, glucagon, arginine, levodopa, GHRH, and macimorelin. Although ITT is considered the gold-standard GH provocative test, it is rarely used nowadays given its hypoglycemic risk, and it is contraindicated in children with seizure disorders and neurodevelopmental delay [16]. Clonidine is a safer alternative and a relatively potent stimulant of GH according to previously published studies [17,18]. Clonidine is a centrally acting alpha-2 adrenergic agonist that increases GH levels by stimulating GHRH and inhibiting somatostatin release from the hypothalamus [19]. Rare side effects of CST include drowsiness and hypotension. Despite its many advantages and safety, glucagon has been less often studied in children as a GH provocative agent to date as compared to CST [20,21]. The exact mechanism of how glucagon stimulates GH release is unknown. Proposed mechanisms include glucagon stimulating GH release by inducing glycemic fluctuations and an increase in norepinephrine levels via proteolytic glucagon fragments that activate central noradrenergic pathways [22,23]. Although GHRH and arginine are relatively potent stimuli for GH secretion, their cost and availability are significant limitations for their use in most countries, including India. Recently, macimorelin, an oral ghrelin mimetic, was studied as a GH stimulant and approved for diagnosing GHD in adults; however, in pediatric populations, safety and efficacy data are limited [24,25].

Following improvements in immunometric GH assays and changes in the diagnostic cutoff for GHD, few studies have compared the efficacy of different provocative tests, including CST and GST. Additionally, there is a paucity of data on the timing and frequency of blood sampling after GH stimulation tests in existing guidelines and published literature [20,21,26-29]. In the present prospective study, peak and mean GH levels were assessed after both stimulation tests. Peak GH levels following CST were highest at 90 minutes (50.90%), followed by 60 minutes (38.20%) and 0 minutes (9.10%); no peak was observed at 30 or 120 minutes. Morris et al. omitted the 30-minute GH sampling step in their CST protocol. Muster et al. concluded that 30-minute sampling is the least important in CST [26,30]. Two other small studies showed that skipping 120 minutes resulted in acceptable false-positive rates of 1.52% and 1.7%, respectively [26,30]. The study by David Gillis et al. also favored the view that a 120-minute sample is superfluous and that CST could have been safely terminated at 90 minutes [28]. Similar to present results, Galluzzi et al. demonstrated that 3.7% of false-positive results were eliminated by omitting the 120-minute blood sample [27]. On the contrary, Thakur et al. demonstrated that sampling GH at 60 and 90 minutes while skipping other time points resulted in a false-positive rate of 7.7% [29]. Rarely, the GH peak occurs at 0 minutes, even before the GH stimulant is administered. Moreover, a “0” sample helps diagnose GH insensitivity (Laron dwarfism); hence, removing baseline GH sampling would alter the results. Therefore, 0-minute GH sampling is necessary and can be easily obtained at the time of intravenous line insertion [28]. Based on the present and previously published literature, 30- and 120-minute sampling are not required, and 0-, 60-, and 90-minute GH sampling are sufficient to diagnose GHD following CST in all cases of severe short stature without compromising accuracy.

Higher mean peak GH levels after GST were observed at 180 minutes compared to 0, 30, 60, 90, 120, and 150 minutes. A significant percentage of peak GH levels was also observed at 0 minutes (14.50%), 120 minutes (38.20%), and 180 minutes (45.5%), whereas no single GH peak was observed at 30, 60, or 90 minutes. Therefore, it is possible to reduce the frequency of GH sampling to three times and the sampling time to 0, 120, and 180 minutes following GST, at the expense of a 2.2% loss in specificity. On the contrary, Christoforidis et al. showed that reducing the number of GH sampling points to three at 90, 120, and 150 minutes resulted in a false-positive rate of 5.75% [20]. Another study by Georeli et al. demonstrated that 71% of GH peaks occurred at 120 and 150 minutes [21]. However, in the present study, 83% of GH peaks occurred at 120 and 180 minutes. Henceforth, 120 and 180 minutes are the most crucial sampling time points after GST. Importantly, the most robust and independent finding was the optimization of sampling times, with over 98% of GH peaks occurring at predefined time points, allowing safe streamlining of protocols without compromising diagnostic yield.

Both CST and GST demonstrated high diagnostic accuracy for GHD, with AUC values exceeding 0.90, signifying excellent discrimination. While GST exhibited higher specificity (100%) and a slightly superior AUC compared to CST (0.987 vs. 0.914), the difference was not statistically significant. Given the high prevalence of GHD in this study population (74.5%), a diagnostic approach prioritizing specificity, such as GST, may be preferred to reduce false positives and unnecessary interventions. However, CST remains a valuable alternative, particularly in settings where GST is unavailable or contraindicated. Although high sensitivity and specificity were observed for both CST and GST, these metrics should be interpreted with caution, as diagnostic classification in this study was derived from the stimulation tests themselves rather than from an independent biological gold standard. Accordingly, the reported diagnostic performance primarily reflects inter-test concordance rather than external validation. The presence of discordant results, wherein some children failed CST but passed GST, is a clinically meaningful finding that supports guideline recommendations for using two stimulation tests and highlights biological variability in GH secretion. GST demonstrated higher specificity than CST within the study framework, representing relative performance rather than definitive superiority. The perfect performance of the combined testing model reflects the internal consistency of the study design.

The main strength is that this is a prospective study, whereas previous studies conducted in children with short stature were often retrospective. The meticulous history, upper and lower segment ratios, all syndromic features, and BMI were taken into account in the statistical analysis, and all peripubertal children were primed before provocative tests. The bone age and BA/CA ratio were calculated, and all subjects underwent MR imaging of the pituitary. Additionally, all subjects underwent both provocative tests, and the highly accurate ECLIA method was used to estimate GH levels; children with ISS served as the active comparator. The primary limitation of this study is the absence of an independent gold standard for diagnosing GHD, such as the ITT, which was not employed due to safety concerns and limited routine pediatric use. Consequently, GHD was defined using a composite clinical and biochemical framework based on CST and GST responses; therefore, the findings should be interpreted as reflecting inter-test concordance and comparative performance rather than absolute diagnostic accuracy. The relatively small sample size and inclusion of predominantly severe short stature cases may limit generalizability, with diagnostic performance potentially overestimated compared to cohorts with milder growth impairment. Although sex-steroid priming was performed according to Tanner staging, its use in peripubertal children may still influence peak GH responses and contribute to inter-individual variability.

## Conclusions

The diagnostic accuracy of the CST and the GST for diagnosing GHD in children with severe short stature is equally high. The frequency of blood samples may be reduced to three, with sampling times at 0, 60, and 90 minutes after CST, and 0, 120, and 180 minutes after GST, without compromising accuracy. This study should be interpreted as a comparative, paired evaluation of CST and GST using modern GH assays, emphasizing test concordance, feasibility, and clinical performance, rather than a definitive validation of diagnostic accuracy.
